# Comparing Bone Fusion Rates Between Novel Unidirectional Porous Tricalcium Beta-Phosphate and Autologous Bone in Lumbar Lateral Interbody Fusion: A Two-Year Radiographic Outcome Study

**DOI:** 10.7759/cureus.46240

**Published:** 2023-09-30

**Authors:** Hiroshi Kumagai, Toru Funayama, Kosuke Sato, Hiroshi Noguchi, Tomokazu Yoshioka, Masao Koda, Masashi Yamazaki

**Affiliations:** 1 Orthopedic Surgery, Ichihara Hospital, Tsukuba, JPN; 2 Orthopedic Surgery, University of Tsukuba, Tsukuba, JPN; 3 Orthopedic Surgery, Kenpoku Medical Center/Takahagi Kyodo Hospital, Takahagi, JPN

**Keywords:** bone union, spine, artificial bone, β-tricalcium phosphate, llif, lumbar interbody fusion

## Abstract

This retrospective cohort study aims to examine the potential differences in bone fusion between autologous bone and artificial bone in the lumbar lateral interbody fusion at 2two years post-surgery.

The bone fusions performed in 15 cases and at 34 intervertebral levels were compared to assess the differences between the artificial bone, Affinos^® ^(Kuraray Co., Tokyo, Japan), and autogenous bone. Two years post-surgery, we evaluated computed tomography (CT) multi-planar reconstruction images in the coronal and sagittal planes.

One year after surgery, out of the 24 windows, 17 (70.8%) windows transplanted with autologous bones showed bone fusion. Additionally, out of the 38 windows, 18 (47.4%) windows transplanted with Affinos^®^ showed bone fusion. Two years post-surgery, out of the 24 windows, 19 (79.2%) windows transplanted with autologous bones showed bone fusion. Additionally, out of the 38 windows, 30 (79.0%) windows transplanted with Affinos^®^ showed bone fusion, and no difference was observed in the fusion rate at two years post-surgery (*P *= 0.238).

In cases using Affinos^®^ for transplanted bone, the bone fusion rate increased between one and two years. The rate of bony fusion using Affinos^®^ in lateral lumbar interbody fusion (LLIF) cages is at par with that of autologous bone grafts at two years post-surgery. Affinos^®^ is a promising candidate for graft material in LLIF surgery.

## Introduction

Lateral lumbar interbody fusion (LLIF) procedures are performed widely to treat various kinds of spinal diseases, including degenerative kyphoscoliosis, spinal canal stenosis, and ossification of the spinal ligaments [1-4].

The LLIF cage has a large footprint for strong interbody correction and stability [5]. Additionally, a substantial quantity of grafted bone can be implanted in the cage.

There is no consensus on the best material to use for grafts. Collecting autologous bone is the gold standard, but complications associated with iliac bone collection have been previously reported [6-8].

Additionally, a two-stage surgery from the anterior to posterior is often performed, and it may be difficult to concurrently collect local bone [9]. Bone morphogenetic proteins (BMPs) can be used in the United States, but in some countries, BMPs are not considered safe for clinical use [10].

Artificial bone, allogeneic bone, and decalcified bone matrix are often used when it is difficult to obtain a sufficient amount of autologous bone [11,12].

Affinos® (Kuraray Co., Tokyo, Japan) is a β-tricalcium phosphate artificial bone consisting of a novel unidirectional porous structure and a porosity of 57%, in which intercommunicating holes are arranged in one direction [13,14]. Affinos® has the following features: rapid penetration of the structure into the material, sufficient strength to withstand compressive stresses along the direction of unidirectional porosity, and ease of handling foam in specific applications [15,16].

From such structural properties, we hypothesized that Affinos® is suitable for LLIF bone grafting. In our previous study, bone fusion in cages transplanted with Affinos® was 70.9% at one year post-surgery, and subsequent results are unknown [17].

This study aims to examine whether there are differences in bone fusion between autologous bone and artificial bone at two years post-surgery.

## Materials and methods

Ethical approval for the study design was obtained from the Institutional Ethics Review Committee of the University of Tsukuba. Written informed consent was obtained from all individual participants included in the study. 

This study included patients who underwent LLIF at our hospital and were regularly followed for at least two years after surgery. We included 15 patients (six men and nine women). The average age of the participants during the time of surgery was 64.7 years (range 44-81 years). The etiology for surgery was lumbar spinal canal stenosis (10 cases) and degenerative kyphoscoliosis (five cases). The number of intervertebral levels fused was 34, which included one case of one-level fusion, 11 cases of two-level fusion, one case of three-level fusion, and two cases of four-level fusion. The comorbidities were osteoporosis in three cases, diabetes in three cases, and rheumatoid arthritis in three cases (Table 1).

**Table 1:** Patient demographics.

Characteristic	Statistics, *n* = 15
Age range (years)	67.9 (55-81)
Male:female	6:9
Diagnosis, *n*	
Lumbar spinal canal stenosis	10
Degenerative scoliosis	5
Comorbidity, *n*	
Diabetes	3
Osteoporosis	3
Rheumatoid arthritis	2
Number of treated segments	
One-level	1
Two-level	11
Three-level	1
Four-level	2

Surgical procedures

We performed LLIF and posterior decompression, with instrumentation, as one-stage (LLIF was initially performed in a lateral decubitus position followed by a posterior fusion in a prone position on the same day) or staged surgery (LLIF in a lateral decubitus position and the posterior fusion was performed in the following week). In this series, we conducted posterior decompression in all cases. Extreme lateral interbody fusion (XLIF) cages (Nuvasive, San Diego, CA) with a 10° angle were used (except for at one intervertebral level where the cage angle was 8°), and the height was determined by intraoperative trial fitting to range from 8 to 12 mm. The cage had large windows on each side for bone grafting. The eleventh rib and iliac bone, collected during the approach, were used as the graft bone. If there was a shortage, Affinos® was used. Minced local bones (rib and iliac bone) were transplanted to 24 graft windows, Affinos® was transplanted to 38 graft windows, a combination of minced local bones and Affinos® was transplanted to five graft windows, and one window did not receive any transplant.

Radiological evaluation

**Figure 1:**
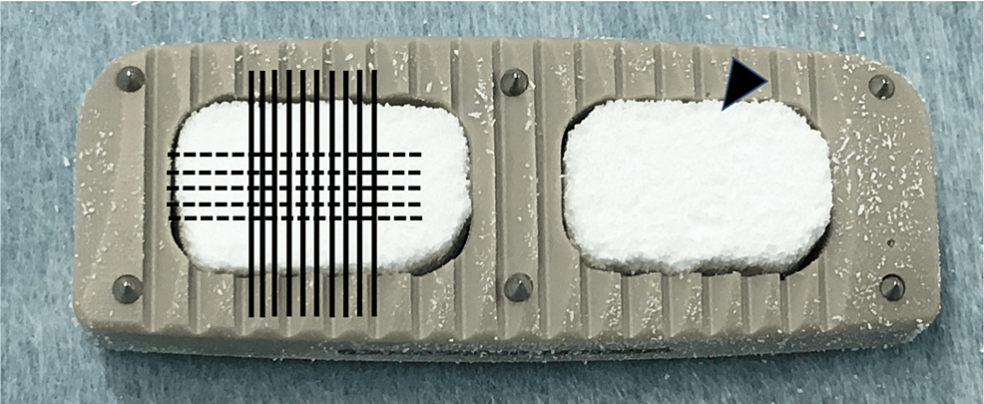
(Solid line) Sagittal view; (dotted line) coronal view; (black triangle) Affinos® inserted in the graft window.

We evaluated the 68 graft windows in the cages at 34 intervertebral levels. To evaluate interbody fusion, CT multiplanar reconstruction coronal and sagittal plane images taken at one and two years post-surgery were evaluated. The coronal reconstructed images were obtained in the plane parallel to the long axis of intervertebral cages, and slices at 2 mm intervals fully covered the anteroposterior diameter of the graft window area in the cage. The sagittal reconstructed images were also obtained in the plane parallel to the short axis of the intervertebral cages, and 2 mm interval slices fully covered the width of the graft window area in the cage (Figure 1). The CT parameters included a window level of 300 Hounsfield unit (HU) and a window width of 2,000 HU.

**Figure 2:**
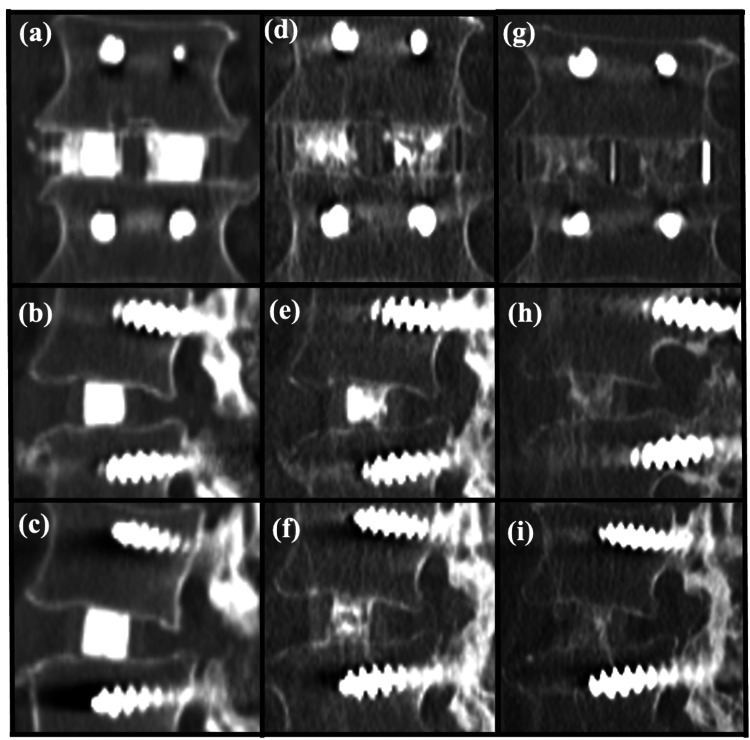
Postoperative coronal and sagittal CT scans. (a) Coronal view, (b) the right side of the sagittal view, and (c) the left side of the sagittal view: CT scans at one year postoperatively. Material resorption and newly formed bone are observed around endplates. (d) Coronal view, (e) the right side of the sagittal view, and (f) the left side of the sagittal view: CT scans at two years postoperatively. Material resorption and bone fusion were observed throughout the graft window area. (g) Coronal view, (h) the right side of the sagittal view, and (i) the left side of the sagittal view. CT, computed tomography

The *intra-window bony fusion* was defined as trabecular bone continuity between the grafted bone within the window of the cage and vertebral endplates in at least one sagittal or coronal slice of CT multiplanar reconstructed images (Figure 2). When the bridging callus was observed outside the cage, it was defined as *extra-cage bony fusion*. The interbody fusion was defined as an intra-window or extra-cage bony fusion in at least one location. 

Three spine surgeons uninvolved in the surgeries assessed the CT images. The inter-rater reliability was examined. The correlation between the rate of bony fusion was assessed. A chi-square test was used to compare the rate of bony fusion between autologous bone and Affinos®, and *P* < 0.05 was considered statistically significant. Analyses were performed using IBM SPSS Statistics version 22.0 (IBM Corp., Armonk, NY).

## Results

The inter-rater reliability had an intraclass correlation of 0.707 and was considered substantial. Thus, the reliability of CT image assessments in this study was acceptable.

One year after the surgery, out of the 24 windows, 17 (70.8%) windows transplanted with autologous bones showed bone fusion. Additionally, out of the 38 windows, 18 (47.4%) windows transplanted with Affinos® showed bone fusion. Out of the five windows, five (100%) windows transplanted with the combination of minced local bones and Affinos® showed bone fusion. Two years post-surgery, out of the 24 windows, 19 (79.2%) windows transplanted with autologous bones showed bone fusion. Additionally, out of the 38 windows, 30 (79.0%) windows transplanted with Affinos® showed bone fusion, and no difference was observed in the fusion rate at two years post-surgery (*P* = 0.238; Table 2). One window that was not grafted did not fuse even after two years.

**Table 2:** Fusion rate (graft window area). POY, postoperative year

Type of graft bone	n	POY1	POY2
Autologous bone	24	17/24 (70.8%)	19/24 (79.2%)
Affinos®	38	18/38 (47.4%)	30/38 (79.0%)
Autologous bone ＋ Affinos®	5	5/5 (100%)	5/5 (100%)
Defect	1	0/1 (0%)	0/1 (0%)

Of the 34 interbody fusions, six did not display intra-window bony fusion and extra-cage bony fusion one year post-surgery. Two years after the surgery, of the 34 interbody fusions, three did not show intra-window bony fusion and extra-cage bony fusion. The interbody fusion rate was 82.4% at one year post-surgery and 91.2% at two years post-surgery (Table 3).

**Table 3:** Fusion rate (interbody fusion). POY, postoperative year

POY1, *n* (%)	POY2, *n* (%)
28/34 (82.4)	31/34 (91.2)

Interbody fusion was not obtained between the three levels in two patients: one patient had severe renal dysfunction, and the other patient with one level in the lower end experienced premature screw loosening after surgery.

## Discussion

The present results showed that Affinos® has a bone fusion rate equivalent to that of autologous bone in LLIFs at two years post-surgery.

Bone fusion is an important concern when performing spinal fusion surgeries. Pseudarthrosis is a serious complication that may require additional surgical interventions. Past clinical trials have shown that the rate of fusion using autologous grafting material ranges from 85% to 97% at one year post-surgery [18-20].

Satake et al. reported that among the 125 segments using allogenic bone in LLIF cages, 69 (55.2%) segments were fused inside the cage based on CT evaluation as done two years postoperatively [21]. Kushioka et al. reported that the bone fusion rate in the cage when using Hap/Col was 19.7%, indicating that the use of artificial bone alone is not sufficient [22].

In terms of artificial bone substitutes, recombinant human bone morphogenetic protein-2 (rhBMP-2) is widely used. The interbody fusion rates using rhBMP-2 via LLIF were 92% in one-level cases and 86% in two-level cases [23]. However, rhBMP-2 is not available in some countries due to concerns about potential side effects, such as hematoma formation and ectopic ossification [24].

Several studies have shown promising results using Affinos® as graft material [25,26].

Sato et al. reported that bone grafting using equal amounts of autologous bone and Affinos® for posterolateral lumbar fusions in a canine model results in bone fusion rates comparable to that of autologous bone grafting [27].

**Figure 3:**
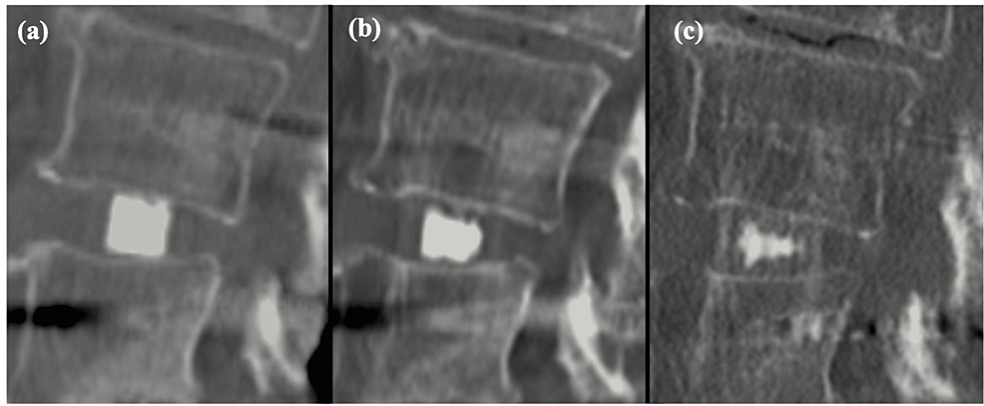
Progression of interbody fusion on sagittal CT scans at (a) seven days, (b) one year, and (c) two years postoperative follow-up. Bone fusion was not obtained one year after the surgery, but bone fusion was obtained two years later.

The results of this study indicate that bone fusion is insufficient at one year post-surgery (Figure 3), necessitating caution for the stand-alone use of Affinos® and in the loosening of the posterior instrumentation [28,29].

A limitation of this study is the small number of scoliosis cases. Artificial bone lacks bone inductive capability, so the bone fusion rate is lower on the convex side, where the contact surface area is smaller compared to the concave side [22]. It is necessary to conduct further experiments to determine if similar results can be obtained when using Affinos®. 

The present results showed that Affinos® has a bone fusion rate comparable to that of the autologous bone in LLIFs.

The use of artificial bone removes the need for separate skin incisions to harvest the bone, thereby reducing complications such as nerve injuries and pain at the bone harvest site.

## Conclusions

In conclusion, in cases of using Affinos® for transplanted bone, the bone fusion rate increased between one and two years. The rate of bony fusion using Affinos® in LLIF cages was not inferior to that of an autologous bone graft at two years post-surgery. Affinos® is, therefore, a promising candidate for graft material in LLIF surgeries.
